# The meanings of consent to the donation of cord blood stem cells: perspectives from an interview-based study of a public cord blood bank in England

**DOI:** 10.1258/ce.2009.009028

**Published:** 2010-03

**Authors:** Helen Busby

**Affiliations:** Institute for Science and Society, University of Nottingham, Nottingham NG7 2RD, UK

## Abstract

This paper explores the perspectives of women who have agreed that their umbilical cord blood may be collected for a public ‘cord blood bank’, for use in transplant medicine or research. Drawing on interview data from 27 mothers who agreed to the collection and use of their umbilical cord blood, these choices and the informed consent process are explored. It is shown that the needs of sick children requiring transplants are prominent in narrative accounts of cord blood banking, together with high expectations for future applications of stem cells. Given this dynamic, a concern arises that the complex and multiple uses of tissues and related data might be oversimplified in the consent process. In conclusion, the positive finding of a commitment to mutuality in cord blood banking among these women is underlined, and its implications for the wider debate on cord blood banking are discussed.

## Background: cord blood stem cell banking

Stem cells isolated from blood or bone marrow are known as haematopoietic stem cells. Stem cells from umbilical cord were first used in transplant medicine in 1988.^1^ Over the next two decades, a scientific consensus was slowly forged that the use of cells from cord blood was an effective alternative to cells from bone marrow in treatments for some malignant and metabolic diseases.^2^

The great majority of cord blood transplants carried out have been of cells from unrelated donors, with a process of tissue typing taking place to ‘match’ donor and recipient cells as closely as possible. This avoids some of the pressures and ethical dilemma associated with using cells from related donors. Following processing and testing, these cells are stored at extremely low temperatures, until they are identified as a good or sufficient match for a patient in need of a transplant. Hence, they can be stored for future use in the treatment of patients with malignant or other serious diseases. National and international registries hold data about these cells, in order that searches can take place for patients as the need arises. A major clinical advantage of using the cells already collected from umbilical cord blood is that it is relatively quick to obtain the cells once a patient needs them.^3^ This contrasts with the situation for bone marrow donors, in which a period of several months typically elapses while a potentially suitable volunteer is contacted and it is confirmed that they are willing and able to donate.

A useful distinction can be made between the private cord banks that operate in the commercial sector and the public cord banks that are supported by public funds. Taking a population approach to health need, public cord banks aim to maximize successful matches for transplants for patients with acute leukaemia and other severe diseases. In contrast, commercial organizations offer a service on demand to parents who can and wish to pay for it generally for their own family's exclusive use. These private cord banks often promote speculation that novel cell therapies *might* be developed that utilize the patients' own pre-deposited cells. Their activities have proved controversial, not least because they promote and could profit from unrealistic expectations about future regenerative therapies. There have also been concerns expressed about possible risks to the mother of the process of cord blood collection. However, there is a lack of clear evidence about this.^4^ Professionals involved in cord blood collection for public facilities argue that with good practice, donors will not be put at risk.^5^ The regulatory stance taken towards such enterprises varies greatly across health-care systems and countries. Clinicians, ethicists and politicians who have objected to private cord blood banking emphasize that solidarity should be the key principle in tissue banking, as has long been the consensus in Europe.^6^ Private and consumer activity in this respect is seen as rupturing that consensus. While the depth and importance of this controversy is clear, it has arguably dominated discussion to the extent that a wider exploration about cord blood banking and public policy has yet to take place. This might include a consideration of parents' involvement in the now extensive public cord blood bank sector. It is to this wider discussion that this paper is addressed.

## What do we know about mothers' views on the collection of cord blood for public cord blood banks?

Very little is known about the perspectives of mothers agreeing to the collection of umbilical cord blood for public cord banks, whether for medicine or for research. Scientific publications predominantly refer to ‘cord blood donors’ only insofar as the variables they present may pertain to the biological quality of cord blood. The question of the views of fathers is largely absent, as are the views of ‘donor children’. (The tentative use of terminology here reflects some fluidity about claims to ‘ownership’ of this biological material: although it is usual practice in the UK to treat this biological material as belonging to the mother, there is also a claim that it ‘belongs’ to the child. Depending on which view is accepted, the mother would be consenting to donate, or agreeing to the collection of the child's cord blood.^7^) Among the few studies published about donors' views is Danzer *et al.*'s study^8^ with women who had donated cord blood. Based on questionnaire data, this is the only publication found in a recently conducted literature review that explored women's views after rather than before the birth. The great majority of Danzer *et al.*'s respondents reported that they would donate again. However, they also ‘indicated having anxiety or objections regarding genetic testing and about the possibility of improper use of donated (umbilical cord blood)’.^8^ Fernandez *et al.* ^9^ conducted a survey of knowledge and attitudes about cord banking among women attending an antenatal clinic in Halifax, Canada. A high proportion of those questioned supported the idea of public cord banking. However, some wanted more information on cord banking and about a quarter indicated that the cord blood bank should not be used to investigate the health of the newborn. It appears from these studies that some of the practices that are considered usual and necessary by cord banks are viewed with some ambivalence by those women whose views have been sought.

Moving to the question of knowledge and expectations surrounding cord blood banking, Fox *et al.* ^10^ conducted a survey of pregnant patients in a New York antenatal facility. They point to knowledge of cord blood therapies being ‘strikingly poor’, and to expectations being very high. Specifically, the great majority of patients in Fox *et al.*'s study believed that cord blood cells have already been used successfully to treat Alzheimer's disease, Parkinson's disease and spinal injury, which is not the case. These recent findings about the beliefs of pregnant women attending a New York clinic may be illuminated by sociological work on the dynamics of expectations in stem cell medicine.^11^ For sociologist Catherine Waldby, the emphasis has been on private cord banking, which she describes as offering ‘a form of popular participation in the open-ended promise of commercial biotechnology’.^12^ However, the view taken in this paper will be that expectations in the possibilities and futures represented by stem cell science may be considered a broader phenomenon, as we can discern in the report of the findings from a large-scale ‘stem cell dialogue’ recently conducted in the the UK.^13^ Thus sociological work on expectations may be relevant to the scientific field more broadly, including activities in the public sector. This paper will draw upon these sociological perspectives in thinking about women's involvement in cord blood donation, as well as on the limited literature on consent referred to above.

As we have seen, little is known about how cord blood donation might be experienced, how the consent process is negotiated, and what it might mean to those involved. Taking inspiration from discussions on empirical bioethics,^14^ and from qualitative research on the process of informed consent,^15^ this paper begins to explore these questions.

## Interviews with women consenting to cord blood collection and ‘banking’ for future public and research use

This paper draws on an empirical case study of a new public cord blood bank in England that is being developed by an established bone marrow donor charity in collaboration with the National Health Service (NHS). The initiative aims to expand the collection and supply of cord stem cells for medical treatment and research, which is already conducted on a limited scale by the NHS National Blood Service. As the likelihood of finding a matched bone marrow donor is lower for non-Caucasian patients than for Caucasians, the new initiative – like most public cord blood banks – has a particular focus on collecting large numbers of donations from an ethnically diverse population. A major aim of the new collection programme is to give thorough information to prospective mothers on the uses and potential benefits of cord blood banking, and to obtain their consent to collect the cells for the public bank.^16^

The author, who is conducting a larger project about multiple perspectives on the ethics of cord blood banking, approached the team to request access to interview women taking part in the new collection programme. Following an extensive discussion of the operational and ethical implications, access was agreed. Once agreed, established conventions for recruitment of interviews were followed: the midwife coordinator sought consent from participants to ‘opt in’ to interviews if they wished to do so by authorizing that their contact details be passed on. The author did not observe any clinical consultations but did observe four of the midwife's presentations to groups of parents who were visiting the hospital. Given the pressures on women during the antenatal period and when attending the hospital, it was decided that speaking with them after the birth was the most appropriate way of conducting these interviews. Forty-five women who were due to give birth at the collaborating NHS maternity hospital over a period of six months consented to the collection of cord blood for the pilot stage of the new programme. This entailed giving their agreement that blood from the umbilical cord could be collected by a midwife with specialist training, who was not part of the team providing clinical care to the mother and baby, if she felt it was appropriate to do so at the time of their delivery. Of these women, 36 also ‘opted in’ to be contacted at a later date about a research interview.

The thirty-six women recruited in this way were contacted for interview at six–eight weeks after their childbirth, regardless of whether the donation was eventually able to go ahead. In three cases, the collection of cord blood had not proceeded due to either a lack of available personnel or clinical considerations at the time of delivery. Twenty-seven interviews were undertaken with women who had consented to cord blood collection, and in five cases husbands or partners were also able to take part in the interviews. The remainder either had moved away from the area (3), could not be contacted (4) or did not wish to take part in the interviews (2). Reasons cited for not taking part in the interviews were pragmatic (lack of time), or a feeling that, as the cord blood could not be collected, there was little to say in an interview about this project. Because this is a small group of patients who might be identifiable from details such as their ethnicity and occupations, these details are not provided for individuals, to whom anonymity was promised. It was agreed by an NHS ethics committee that these interviews could be undertaken. Women registered as ‘cord blood donors’ who had agreed to be contacted were phoned 6–8 weeks after the birth of their babies to ask if they would be willing to be interviewed. Arrangements were then made if they wished to go ahead. The interviews were conducted at the interviewees' own homes, with the exception of one conducted by phone and one at the hospital. All interviews were recorded, with consent, and transcribed. Most of those interviewed had just had their first baby, and the majority held professional jobs, to which they intended to return. The predominant ethnicity of this group according to their own definitions was ‘White British’, but the sample also included women from European and Black Afro-Caribbean groups.

A topic guide for these interviews included questions designed to illuminate the knowledge, expectations, concerns and experiences of these women in relation to cord blood banking. The analysis focused on these areas and in particular on an exploration of the ethical and practical rationales presented for donating to the public cord bank. A data matrix was used to summarize key aspects and themes from the data. Techniques from ‘grounded theory’ approaches were used to open up an exploration of what consent means in this context.^17^

## The recruitment process for women agreeing to collection of cord blood

Following the implementation of the Human Tissue Act (2004), the obtaining of consent is a formal requirement for the collection or ‘procurement’ of human tissues in England, Wales and Northern Ireland.^18^ This consolidates a shift from a regulatory approach in which tissues obtained in clinical contexts were sometimes regarded as ‘abandoned’, to one in which consent from the patient is seen as central. This qualitative research focused on exploring donors' narratives, rather than scrutinizing the effectiveness of information-giving or compliance with formal consent processes. However, it was hoped that exploring and describing the women's experiences would be informative for the cord bank team as they reviewed their practices and moved forward with their project.

The primary means for providing information to potential donors were information leaflets made available at various points in the hospital's maternity services, and presentations on the project after the regular ‘labour ward tour’, which women may attend towards the later stages of their pregnancy, often accompanied by their partners. The information leaflet provides basic information about the process of collection and of consenting or ‘opting in’ to participation in the project. Written information specifies that consent is to collection of cord blood and blood samples, testing of cord blood and samples, use of relevant medical data from the maternity unit, the possibility of clinical feedback from testing in some cases, use of cord blood for a public cord bank, in the cell therapy laboratory, and in ethically approved research. It is stated that cord blood only meets the threshold quality standards currently used in transplant medicine in less than half the cases where collection takes place. The possibility of the remaining cord blood being used for research is therefore also explicitly referred to in the introductory leaflet.

During her presentation, the midwife coordinator gives a layered and historical account of the rationale for the collection of cord blood cells. The presentation takes the form of an account of the setting up of national and international bone marrow registers, and of the emergence of umbilical cord as an alternative source of stem cells for transplants. An indication is given of the range of diseases that are, sometimes, treated with these cells. The recipients of transplants, often children with acute leukaemias or severe blood disorders, have a central place in this narrative, and those who died waiting for a transplant are also referred to. The project's cell bank and research laboratory, and their aims, are described in terms of increasing the availability of matched stem cell units for transplant and developing future treatments. Thus the project that is presented crosses the scientific divide between conventional transplant medicine/haematology and regenerative therapies, and encompasses both these possible futures. A promissory dynamic is evident here, but one that is different from the individualized one that has been described in relation to private cord blood banks.^19^ The narrative that emerges is a powerful one that merges together the missing futures for the children who did not reach adulthood, the futures regained for children who were recipients of successful transplants and the future needs of unknown children and adults. The promise that is being constructed refers both to established transplant treatments that are life saving for some children with severe diseases, and to possibilities of new regenerative therapies in the future. While the project information refers to the possibility of cord blood being used in research, the headlines of patient leaflets, press releases and the midwife's presentation all refer to the double ‘gift of life’ of a newborn who might save the life of a sick child. This is echoed in the following phrase on the cover of the information leaflet for women invited to donate: ‘Your Birth Day gift…Helping to save a life’.

The collection procedure is also explained by the midwife coordinator, who conveys with conviction the commitment that collection would only take place if she was assured that it would in no way compromise the care of mother and baby. The potential for transformation from donated cells is contrasted with the usual alternative to donation (in this and many hospitals), which is disposal of tissues that are designated as clinical waste. The mother also agrees to donate blood samples for testing, and for any tests to be carried out on the blood sample or the cord blood itself that may be in the interests of recipients. She must consent to being contacted if any ‘positive results’ are found that may have implications for her or her baby's health – and is advised that she may not take part if she does not wish to be contacted about these. Thus she is agreeing to the use of her own and her child's biological material and related data in a number of ways, and over an unspecified period of time. Finally, the mother agrees to re-contact at six months for a second blood test and a health questionnaire.

## Consent and the framing of possibilities

All of the women interviewed said that they had discussed the cord blood collection with the midwife coordinator and had been given written information before giving their consent, thus meeting the formal ethical requirements for a project of this kind. Some had not heard of the possibility of cord blood banking before:

‘I'd never even heard of cord blood banking. To me it was just something, I just assumed that like the placenta, it was just thrown away straight after the birth.’ (Interview 23)

While a few of the early donors were members of the hospital professional community, the majority had become involved with the project on the basis of seeing the project leaflet or hearing about the project during their labour ward tour. Still, they were aware of the hospital's status as a teaching hospital with high levels of research activity, and especially of its high reputation in the fields of midwifery and obstetrics. It was evident that many of these donors approached the invitation to participate in this light. Consenting to the cord blood collection, then, was bound into the relationships with staff at the maternity unit services. However, most viewed the cord blood donation process as ‘straightforward’, in that no additional appointments or major interventions were required:

‘It didn't involve a lot from us, because it was something that was valuable for… valuable for us in terms of, you know, we got to feel like we were doing something useful and helpful and the possibly that maybe if we needed it [in] the future we might be able to access it. And …something that would go to waste would be helpful for other people.’ (Interview 24)

The suggestion that waste disposal would be the alternative destiny for umbilical cord, voiced in these two short extracts, was a common one. The posing of these stark contrasts between waste disposal and biological potential is part of the compelling nature of these narratives. This discourse has some similarities with language sometimes used about the sourcing of tissues and cells from women attending *in vitro* fertilization and abortion clinics.^20,21^ Yet while researchers working in those contexts found ambivalence and contest around these assumptions among donors, here the idea of cord blood as clinical waste seemed to be shared to a great extent by these mothers and their midwives.

Participants in the cord blood bank have to hold in the balance the possibilities of ‘saving a life’ for a patient who needs a transplant, and of the cells being used in the laboratory. Their particular sample might be used to test a laboratory process or, perhaps, the cells derived from it might be used eventually in the development or testing of new cell therapies. Mothers, and partners when present at the interviews, were asked about any concerns they might have regarding the use of the cord blood. Some did comment on risks that arise when tissues are used for research, and when personal data are kept in this context. A few indicated that they were aware of stories or scandals about blood or organs being sold. One partner expressed his concerns about the possibility that animals might be used in research related to cord blood stem cells in the future – but stated that he had not wished to take up too much time discussing these problems or raising controversial issues at the midwife's presentation. This hints at the ways in which ‘etiquette’ in a clinical context might militate against a robust discussion of research with people who are part of a clinical team caring for one's family. Notwithstanding these doubts, they (all) indicated, however, that they had made a decision to entrust the project with the responsible use of the cord blood cells:

‘Well I will leave it for the medicine and the scientists…I hope it will do all the good.

HB: So, if they do research to understand more about the cells?

Whatever it takes, just take you forward. So I'm fine with that.’ (Interview 18)

There is a sense that the hopeful and optimistic stance often taken in the course of these interviews may deflect participants from dwelling on the possibility of subsequent use of information in this context: as explained above, mothers are informed by the cord bank of the possibility of feedback of information that might be clinically relevant for them, or their children. In addition, it is likely that new tests will in future be introduced for donated cells to minimize the risks of using them in patients/recipients. This dimension does not yet appear to have been sufficiently considered. One outcome of this research therefore was a recommendation to the cord blood bank that the arrangements for feedback to mothers from clinically relevant tests be clarified, and that the implications of such tests for donors be kept under review. The phenomenon of tension between donors' and recipients' interests in relation to screening of donated tissues is by no means exceptional. However, it has emerged in these interviews that there is potential for the intense needs of recipients to overshadow interests of donors in discussions about donation for transplant medicine and stem cell research.

## Discussion: the multiple meanings of consent to the donation of umbilical cord blood

This paper has drawn on a small set of exploratory interviews conducted as part of a larger study about multiple perspectives on cord blood banking, and no claims are made for generalizability of these findings. A positive feature of the approach taken here is that women were interviewed after the birth, but sufficiently close to the time of donation for a detailed discussion to be held about their involvement with the cord blood bank. This is in contrast to the few published studies of women's views on cord blood banking, which have mostly been undertaken before the birth. However, it is clear that the practicalities of caring for a baby of a few months old may also constrain the possibility of fuller consideration of the implications of donating. Different accounts might emerge if donor mothers were re-interviewed. In addition, the perspectives of children might be considered using methods such as those deployed in the Avon Longitudinal study of parents and children.^22^ A longitudinal approach to researching the perspectives and interests of mothers and children would be informative as public cord blood projects of this kind become established in the longer term.

Drawing on interviews with mothers, it has been emphasized that consent in this case is only partially ‘informed’: this is in part because, as in other emerging fields of science where basic and translational research is unfolding, it is not possible for researchers to state in detail what research will be undertaken. Women are invited to agree to authorize the collection of the cord blood, and to agree that the cord bank will in effect be the custodian of the donation for good in the future. The invitation to consider donating cord blood cells thus calls them and their partners into an uncertain future. This uncertainty extends to the use of data from the donated material. The ambiguity about the uses of information in this context – that may for example arise from the testing of the cord blood for the benefit of the recipient – is an important ethical issue for public cord blood banks to address in the future. This relates particularly to the possible implications of such data for ‘donor’ children, who are implicated but too young at this time to be asked about their views.

In this paper, the informed consent process is also seen as a space in which midwives and parents develop narratives about the possibilities associated with the use of cord blood. As with traditional blood services, the initiative builds on the understanding that everyone is potentially dependent on transplant medicine, so that any child or adult may need help from a public cord blood bank. Donors to the cord blood bank also approached this with great interest in the future possibilities of regenerative medicine. In conclusion, while much of the public debate about cord blood banking has focused on the problems associated with the private storage of cord blood stem cells, this study suggests that parents may also be profoundly interested in the future provision of stem cell banking within an ethic of mutuality. Further and larger scale work will be needed to explore the extent, limits and implications of this support for public cord blood banking.

## References

[1] Gluckman E, Broxmeyer HE, Auerbach AD, Hematopoietic reconstitution in a patient with Fanconi's anemia by means of umbilical-cord blood from an HLA-identical sibling. N Engl J Med 1989;321:1174–8 2571931 10.1056/NEJM198910263211707

[2] Rubinstein P. Why cord blood? Hum Immunol 2006;67:398–404 16728260 10.1016/j.humimm.2006.03.015

[3] Barker JN, Krepski TP, DeFor T, Davies SM, Searching for unrelated donor hematopoietic stem cell grafts: availability and speed of umbilical cord blood versus bone marrow. Biol Blood Marrow Transplant 2002;8:257–60 12064362 10.1053/bbmt.2002.v8.pm12064362

[5] Armitage S, Warwick R, Fehily D, Navarrete C, Contreras M. Cord blood banking in London: the first 1000 collections. Bone Marrow Transplant 1999;24:139–45 10455341 10.1038/sj.bmt.1701881

[6] http://ec.europa.eu/european_group_ethics/docs/avis19_en.pdf.

[7] Gunning J. Umbilical cord blood: banking and clinical application. Law Hum Genome Rev 2004;20:217–25 15544151

[8] Danzer E, Holzgreve W, Troeger C, Attitudes of Swiss mothers toward unrelated umbilical cord blood banking 6 months after donation. Transfusion 2003;43:604–8 12702181 10.1046/j.1537-2995.2003.00375.x

[9] Fernandez CV, Gordon K, Van den Hof M, Knowledge and attitudes of pregnant women with regard to collection, testing and banking of cord blood stem cells. Can Med Assoc 2003;168:695–8 PMC154914 12642424

[10] Fox N, Stevens C, Ciubotariu R, Umbilical cord collection: do patients really understand? J Perinat Med 2007;35:314–21 17511596 10.1515/JPM.2007.084

[11] Brown N, Kraft A. Blood ties: banking the stem cell promise. Technol Anal Strateg Manag 2006;18:313–27

[12] Waldby C. Umbilical cord blood: from social gift to venture Capital. BioSocieties 2006;1:55–70

[13] Battachchary D. Stem Cell Public Dialogue Final Report for BBSRC and MRC. British Market Research Bureau 2008. See http://www.mrc.ac.uk/Sciencesociety/Publicinvolvement/Consultations/Stemcelldialogue/index.htm (last checked 13 August 2009)

[15] http://www.childrenshospital.org/research/Site2030/Documents/KatzFoxReport_2004.pdf.

[16] Duffy T, Querol S, Dennes W, The Kingscord model: a public cord blood collection service. Br J Midwifery 2009;17:306–13

[17] Strauss A, Corbin J. Basics of Qualitative Research: Grounded Theory Procedures and Techniques. London: Sage, 1990

[18] http://www.hta.gov.uk/licensingandinspections/sectorspecificinformation/stemcellsandcordblood.cfm.

[19] Brown N, Kraft A, 2006 Op. cit, note xi

[20] Kent J. The fetal tissue economy: from the abortion clinic to the stem cell laboratory. Soc Sci Med 2008;67:1747–56 18945530 10.1016/j.socscimed.2008.09.027

[21] Parry S. (Re)Constructing embryos in stem cell research: exploring the meaning of embryos for people involved in fertility treatments. Soc Sci Med 2006;62:2349–59 16289740 10.1016/j.socscimed.2005.10.024

[22] Goodenough T, Williamson E, Kent J, Ashcroft R. ‘What did you think about that?’ Researching children's perceptions of participation in a longitudinal genetic epidemiological study’. Children Soc 2003;17:113–25

